# Psychological wellbeing and its associated factors among older adults attending daycare centers in Kathmandu, Nepal: A cross-sectional study

**DOI:** 10.1371/journal.pone.0353748

**Published:** 2026-07-15

**Authors:** Rekha Timalsina, Madhusudan Subedi, Nima Maharjan, Bigya Shah, Pabitra Adhikari

**Affiliations:** 1 Patan Academy of Health Sciences, School of Nursing and Midwifery, Lalitpur, Nepal; 2 National Institute on Ageing and Health (NIAH), Kathmandu, Nepal; 3 Patan Academy of Health Sciences, School of Public Health, Lalitpur, Nepal; 4 Patan Academy of Health Sciences, School of Medicine, Lalitpur, Nepal; 5 Ageing Nepal, Kathmandu, Nepal; University of Toronto, CANADA

## Abstract

Psychological wellbeing (PWB) is a vital aspect of healthy aging, encompassing overall functioning, life satisfaction, and the ability to manage life’s challenges. It is shaped by various socio-demographic, financial, and health-related factors. However, evidence on these associations among older adults in Nepal is limited. Accordingly, this study aimed to identify the level of PWB and its associated factors in this population. A cross-sectional analytical study was carried out among 300 older adults attending daycare centers in Kathmandu, Nepal. The respondents were selected using quota sampling based on the daycare centers. Subsequently, face-to-face interviews were conducted to collect data using structured interview schedules covering socio-demographic, financial, and health-related characteristics, as well as the Ryff PWB Scale. The collected data were then entered into Epi data software and analyzed using SPSS version 16. First, descriptive statistics were computed to summarize the sample characteristics and PWB, and subsequently, chi-square tests, and bivariate and multivariate binary logistic regression analyses were performed to examine the associations between the independent variables and PWB. Bivariate analysis shows that sex, marital status, offspring status, literacy status, former employment status, current employment status, pension status, and receipt of the old age allowance (OAA) were significantly associated with PWB. However, in multivariable logistic regression analysis, marital status, literacy status, former employment, and receipt of the OAA were significantly associated with PWB. Married older adults living with a spouse (*AOR* = 1.75, 95% *CI*: 1.02–3.02), literate individuals (*AOR* = 2.12, 95% *CI*: 1.18–3.82), and those with prior employment (*AOR* = 3.94, 95% *CI*: 1.36–11.36) had higher odds of high PWB, with p-value < .05. In contrast, OAA recipients had lower odds of high PWB (*AOR* = 0.47, 95% *CI*: 0.23–0.96, *p* = .037). Age, sex, and pension status were not significantly associated with PWB in the adjusted model. In conclusion, marital status, literacy status, former employment, and receipt of OAA were the factors associated with PWB. A holistic approach is recommended, including targeted interventions for illiterate older adults; those who are divorced, separated, unmarried, or widowed; those without formal employment history; and OAA recipients.

**Trial Registration:** Not applicable. The authors clarify that this study employed a cross-sectional design, with data collected and analyzed at a single time point, and therefore it does not constitute a clinical trial.

## Introduction

The population of older adults aged 60 years and above is increasing globally [1], including in the Asian region [2] and Nepal [3]. This trend is primarily driven by declining fertility, increased youth migration, and improved life expectancy [2,4]. Consequently, it poses significant challenges for social, economic, and healthcare systems [2,4]. As people age, they experience various life transitions that can affect mental well-being. These include managing chronic illness, coping with the loss of loved ones [5], caring for dependent spouses, experiencing reduced income or a loss of purpose after retirement, facing adversity, and declines in intrinsic capacity and functional ability [6]. Although many older adults adjust successfully, some experience persistent grief, social isolation, or loneliness [5–7]. In addition, they may face family adjustment challenges [6], financial dependence, elder abuse [6,7], poor living conditions, ill health, or limited access to quality support and services [7]. Consequently, these psychosocial challenges contribute substantially to psychological morbidity among older adults and are also expected to increase disability, cognitive decline, and suicide risk [6]. Despite these challenges, aging should not be viewed solely as a period of decline [8]. Evidence suggests that many older adults experience positive psychological well-being [PWB] through resilience, emotional regulation, social connectedness, wisdom, and a sense of purpose in life [8]. Older adults frequently demonstrate an enhanced ability to manage negative emotions, focus on positive experiences, and adapt to adversity using accumulated life experience and refined priorities [8]. Furthermore, a sense of meaning and purpose has been linked to better physical and psychological health outcomes [8].

In this context, mental well-being provides an important framework for promoting mental health and managing psychological conditions among older adults [9]. Specifically, mental health is defined as a state of well-being in which individuals can manage life’s stresses, realize their abilities, work and learn effectively, and actively participate in the welfare of their communities [10]. Overall, mental health is fundamentally important for overall well-being [10]. Similarly, Ryff [11] conceptualized psychological wellbeing (PWB) as a multidimensional construct encompassing six key components: self-acceptance, positive relations with others, autonomy, environmental mastery, purpose in life, and personal growth. Together, these dimensions reflect an individual’s overall functioning and optimal psychological health.

PWB and related mental health outcomes among older adults are influenced by multiple socio-demographic, personal, and social factors. These include age [12–15], sex [12,16–19], and marital status [12,13,16,17], education level [12,16–20], employment status [12,18,19], and income and financial support [17–19] as the important determinants of PWB. Moreover, ethnicity and family type [12,19], living arrangements and household composition [14,15,21], social engagement including communication with friends, enacted social support, and social integration [18,21], perceived health and comorbidities [13,17], and relationships with children and spousal companionship [16] are important factors associated with PWB. In particular, in collectivist cultures, family structure, caregiving roles, and spousal companionship are closely linked to the psychosocial well-being of older adults [15].

These associations have been observed globally, among older adults in Europe (Spain [13]; Ukraine [21]), Africa (Ghana [18]; Nigeria [12]), and Asia (Malaysia [15]; Bangladesh [17]; India [14,20]; Bhutan [16]; and Nepal [19]). Nonetheless, most existing studies have focused primarily on psychological distress and mental illness rather than on positive PWB, and have largely been conducted among community-dwelling older adults or those residing in institutional settings. Furthermore, coping mechanisms and approaches to maintaining mental well-being vary across cultural contexts [9]. Older adults attending daycare centers might also face a unique environment that may influence PWB differently from other living arrangements. Consequently, findings from community and institutional settings may not be directly applicable to the older adults attending day care centers. In the Nepalese context, Timalsina et al. [22] identified several factors associated with PWB among older adults, including self-efficacy, resilience, perceived social support, social connectedness, spirituality, and loneliness. Despite these findings, evidence remains scarce regarding the influence of socio-demographic, financial, and health-related characteristics on PWB among older adults attending daycare centers. Accordingly, prior evidence emphasizes the need for future research examining the associations between mental well-being and contextual factors such as access to care, coping strategies, and cultural context [9]. In addition, socio-demographic factors including sex [9,22], income [9], ethnicity, education, household composition, and living conditions, along with comorbidities [22], should be investigated to identify older adults at risk. Collectively, these findings can inform the development of targeted and tailored interventions for aging populations [9,22]. Taken together, these gaps highlight the need for further research examining the determinants of PWB among older adults attending daycare centers in Nepal. Therefore, this study aimed to identify factors associated with the level of PWB, including socio-demographic variables (age group, sex, marital status, offspring status, literacy status, and family type), financial-related variables (former and current employment status, receipt of a pension, financial support from offspring, and old age allowance [OAA]), and health-related variables (presence of chronic diseases, perceived overall physical health status, and ability to perform activities of daily living). Thereby, the findings might be useful to address an important gap in the existing literature.

## Materials and methods

### Study design

A cross-sectional analytical study was conducted among older adults attending daycare centers in Kathmandu, Nepal. Although the same dataset was previously analyzed to examine psychosocial and spiritual factors associated with PWB using a cross-sectional path analysis approach [22], the present study, in contrast, focuses specifically on the association of socio-demographic, financial, and health-related characteristics with level of PWB.

### Setting, and study population

The study population consisted of 1,737 older adults across 14 daycare centers, as previously described by Timalsina et al. [22]. The inclusion criteria comprised older adults aged 60 years or above who attended the daycare centers, consented to participate, could communicate effectively, and were capable of recalling and discussing aspects of their PWB. Older adults with severe comorbidities, psychiatric conditions, or other mental health problems that could affect communication or participation were defined as exclusion criteria. This was based on information provided by participants and center authorities, as medical records were not available during routine visits or at the centers. Importantly, these eligibility criteria were based on functional and clinical conditions affecting participation and were independent of participants’ self-reported perceived health status. However, none of the older adults met these exclusion criteria during the study period. Additionally, older adults who declined at any stage were excluded. Consequently, of the 316 older adults approached by research assistants [RAs], 16 refused participation, resulting in a non-response rate of 5.1%.

### Sample size

This study is part of a larger path-analytical project on PWB. In addition, the project also aimed to investigate 14 socio-demographic, financial, and health-related factors in relation to the level of PWB. In the present paper, these variables were analyzed to assess their association with PWB among older adults within the same study framework. To ensure adequate statistical power and stable estimates for the multivariable analyses, a total of 300 respondents were recruited through a non-probability sampling approach. This sample size aligns with the recommendation of Bujang et al. [23], who suggest that 300 respondents are sufficient for multivariable analyses involving non-random sampling and adjustment of potential confounding variables.

Although 14 variables were initially considered, only seven were retained in the multivariable logistic regression model based on bivariable analysis results. A post hoc sample size adequacy assessment was conducted to evaluate whether the available sample was sufficient for the current analytical objective. This assessment was based on the observed outcome proportion (49.7%) reported in the results section of this paper, and Peduzzi et al.’s [24] formula for logistic regression (n = 10 × k/p), where k = 7 represents the number of independent variables and *p* = 0.497 is the proportion of the less frequent outcome. The required sample size was estimated at 141, which increased to 148 after accounting for a 5.1% non-response rate from this study. However, the final dataset included 300 respondents. This assessment was performed for justification purposes only and did not involve any modification of the original sample size. Overall, the available sample was considered adequate for the multivariable analyses.

### Sampling technique

Considering time, financial, and logistical constraints in covering all 14 daycare centers in Kathmandu, a single-stage cluster sampling procedure was used to select study sites. First, six daycare centers (A-F) were randomly selected from the total of 14 using a random number generator. Within the selected centers, non-probability quota sampling approach was applied, as described in a previous publication [22]. However, respondents were recruited based on the availability of eligible individuals during data collection period. Consequently, the distribution of respondents across centers was disproportionate relative to their population sizes: Center A (total population = 443; n = 104), B (total population = 120; n = 35), C (total population = 122; n = 54), D (total population = 150; n = 31), E (total population = 85; n = 30), and F (total population = 149; n = 46).

### Data collection instruments

The instruments used in this study consist of socio-demographic, financial, and health-related characteristics, and PWB scale.

***Questions related to socio-demographic, financial, and health-related characteristics.*** This section included questions on respondents’ socio-demographic, financial, and health-related characteristics. Socio-demographic and financial characteristics included age, sex, marital status, having offspring, literacy status and educational level, family type, employment status, pension status, financial support from offspring, and receipt of old-age allowance. Health-related characteristics included presence and type of chronic diseases, self-rated overall health status, and ability to perform activities of daily living.

***Psychological Wellbeing Scale.*** The PWB of older adults was assessed using the Ryff PWB scale [25]. This scale comprises 42 items, each rated on a six-point Likert scale, with 1 indicating “strongly disagree” and 6 indicating “strongly agree.” The scale is structured around six dimensions, each containing seven items: Autonomy, Environmental Mastery, Personal Growth, Positive Relations with Others, Purpose in Life, and Self-Acceptance. As an illustration, one item on the scale is: “I think it is important to have new experiences that challenge how you think about yourself and the world.” Notably, the scale includes an equal number of positively and negatively worded items (21 each), with the negatively worded items reverse-scored before computing the total score. The total PWB score spans from 42 to 252, where higher values denote higher PWB. For analytical purposes, in the absence of established clinical cut-off values for PWB as conceptualized by Carol D. Ryff’s PWB model, PWB scores were dichotomized using median split approach into low (≤ Median), and high (> Median), consistent with approaches reported in prior literature by Fergus et al. [26]. Previous studies have demonstrated that Ryff’s PWB scale exhibits satisfactory reliability and reasonable factorial validity among middle-aged and older adult populations [27].

For the present study, the Ryff PWB scale [25] was translated into Nepali language following the six-step process outlined by Borsa et al. [28]. To ensure both cultural relevance and clarity, the translated version was pretested with 43 older adults at two randomly selected daycare centers (‘G’ and ‘H’) in Kathmandu, consistent with the recommendations of Perneger et al. [29]. During this pretesting phase, RAs carried out cognitive interviews and one-on-one debriefings to examine respondents’ comprehension and interpretation of each item. Furthermore, RAs employed behavioral coding during interviews to evaluate respondents’ responses for hesitations, questions, or any difficulties. As a result, these procedures confirmed that all items were clear and required no modifications. Subsequently, reliability analysis based on the pretest sample yielded a Cronbach’s alpha of 0.71, and these pretest data were not included in the main study. When reliability was recalculated using data from the 300 respondents in the primary study, the internal consistency was found to be acceptable (*α* = 0.72).

### Data collection techniques

Following a formal discussion with the research team, two RAs with bachelor’s degrees in nursing and prior data collection experience were recruited and trained. Data were collected from 3 September to 2 October 2024 through face-to-face interviews using a structured Nepali-version interview schedule. The trained RAs collected data in the selected daycare centers (A–F) in coordination with center authorities, and interviews were carried out based on availability of respondents. Each interview lasted approximately 45–60 minutes. The research team conducted random supervisory visits throughout the data collection period to ensure quality control and adherence to procedures. After each interview, the RAs reviewed questionnaires for completeness and, where necessary, followed up with respondents (with informed consent) to obtain missing information.

### Ethical approval and informed consent

All study procedures were conducted in accordance with the ethical principles outlined in the Declaration of Helsinki and complied with relevant national regulations. Accordingly, formal written administrative approval was obtained from the Social Security Department of Kathmandu Municipality and the authorities of each daycare center. Subsequently, ethical clearance was granted by the Ethical Review Board of Nepal Health Research Council (Reference No: NHRC 313/23 August 2024). Ethical procedures were strictly followed throughout the study. Specifically, written informed consent was obtained using informed consent form (See **S4 File.pdf**), and the privacy and confidentiality of all respondents attending the daycare centers were ensured. Although some older adults were illiterate, they were able to provide written informed consent by signing or providing a thumbprint on the consent form. Moreover, any emotional distress experienced during interviews was carefully managed. In addition, relevant health information was provided, and respondents’ concerns were consistently acknowledged and respected throughout the study. Details of the ethical procedures are reported in our previous paper by Timalsina et al. [22].

### Statistical analysis

Completed interview schedule was reviewed by the research team to ensure completeness and consistency. Data were then edited, categorized, and manually coded. To enhance data accuracy, double data entry and data cleaning were performed using Epi data software. Statistical analyses were then carried out using SPSS version 16. Descriptive statistics, including frequencies, percentages, medians with interquartile ranges, and means with standard deviations were applied to summarize the respondents’ characteristics and PWB. Chi-square test was also performed (**S1 Table****. Association Between Different Independent Variables and Level of Psychological Wellbeing in S1_File).**

Prior to analysis, variables were coded based on their distribution and suitability for logistic regression. Some variables were originally binary (e.g., sex, having offspring, literacy status, employment status, pension status, and old-age allowance), while others had multiple categories (e.g., age group, family type, marital status, educational level, and perceived overall health status). Due to sparse cell counts and violations of events-per-variable (EPV) assumptions, marital status, educational level, and perceived health status were subsequently collapsed into binary categories to ensure model stability and interpretability, allowing stable estimation of adjusted odds ratios.

*Clustering Effect Assessment.* Because participants were recruited from multiple daycare centers, clustering effects were assessed prior to regression analysis. Intraclass correlation coefficient (ICC) was estimated using a one-way random effects ANOVA approach following Shrout and Fleiss [30]. The design effect [DEFF] was calculated using formula: DEFF = 1 + (k − 1) ICC as recommended by Alimohamadi and Sepandi [31], where k represents the average cluster size. The results are presented in **S3 Table****: Clustering Effect Assessment (One-way ANOVA Approach) in S3_File.**

For logistic regression analysis, all variables were ultimately coded into dichotomous forms. Socio-demographic variables included age group (≤72 years vs > 72 years; categorized based on the median age), sex (male vs female), marital status (married and living with spouse vs unmarried/divorced/widowed/separated), having offspring (yes/no), literacy status (literate vs illiterate), and type of family (joint/extended vs nuclear). In addition, financial-related variables were included, such as former and current formal employment status (employed vs never employed), receiving pension or social security benefit from employers (yes/no), receiving financial support from offspring (yes/no), and receiving the government OAA (yes/no). Health-related variables were also considered, including the presence of chronic disease (yes/no), perceived physical health status (good/very good vs very poor/poor/fair), and the ability to perform activities of daily living (ADLs) (yes/no). Finally, the dependent variable, level of PWB, was dichotomized into high PWB (1) and low PWB (0), based on previously established categorization criteria [26]

Before conducting multivariable logistic regression, key assumptions were assessed. These included independence of observations (i.e., no duplicate responses) [32], identification of highly influential outliers [32,33], and adequate number of EPV (at least 10 outcomes per binary category) [32], and absence of multicollinearity among independent variables [32,33]. Multicollinearity was evaluated using correlation analysis (**S2 Table****. Correlations of Different Independent Variables with PWB in S2_File)**, variance inflation factor, and tolerance (Note of Table 3 and S2 Table in Supplementary File 1). Subsequently, bivariable logistic regression analyses were performed to examine the association between each independent variable and PWB. Variables with a p-value ≤ 0.25 were considered candidates for inclusion in the multivariable model to minimize the risk of excluding potential confounders [32].

In addition to statistical significance, variable selection for the multivariable model considered EPV recommendations of 10–20 events per covariate to avoid model overfitting [32]. Accordingly, some variables were excluded because of sparse cell counts and unstable estimates (having offspring, current employment status, and ability to perform ADLs) or because they were not statistically significant in bivariable analyses (family type, presence of chronic disease, perceived overall health status, and receipt of financial support from offspring). The final multivariable logistic regression model included age group, sex, marital status, literacy status, former employment status, receipt of pension, and receipt of OAA. Adjusted odds ratios (AORs) with 95% confidence intervals were reported, and statistical significance was set at p ≤ 0.05.

## Results

### Socio-demographic, financial, and health-related characteristics

The study included 300 respondents aged 60–94 years. Baseline sociodemographic characteristics have been reported in previous publication [22]. Table 1 presents that the highest proportion of respondents had no history of formal employment (79.7%) and were not currently engaged in formal work (97.3%). Regarding pension benefits, 87.7% did not receive social security benefit from employers. Financial support from offspring was reported by 66.3% of respondents, and 57.3% were beneficiaries of government-provided OAA. In terms of health, 53.0% rated their overall health as fair, and 98.3% were independent in activities of daily living (Table 1).

**Table 1:** Respondents’ financial and health-related characteristics *N* = 300.

Variables	Frequency (n)	Percentage (%)
**Former Employment Status** ^**a**^		
Never employed	239	79.7
Ever employed	61	20.3
**Receiving Pension (Social Security Benefit from Employers)**		
Yes	37	12.3
No	263	87.7
**Current Employment Status** ^**b**^		
Employed	8	2.7
Not employed	292	97.3
**Receiving Financial Support from Offspring**		
Yes	199	66.3
No	101	33.7
**Perceived Overall Health Status** ^**c**^		
Very Poor	5	1.7
Poor	27	9.0
Fair	159	53.0
Good	94	31.3
Very Good	15	5.0
**Able to Perform Activities of Daily Living**		
Yes	295	98.3
No	5	1.7
**Receiving old age allowance (Social Security Benefit) from the Government**		
Yes	172	57.3
No	128	42.7

*Note.* ^a^: Former employment status: Ever been engaged in paid formal employment. ^b^: Current employment status: Engagement in paid formal employment at the time of data collection. ^c^: Self-reported health status among eligible respondents who were able to communicate and participate.

### Level of psychological wellbeing

Table 2 reveals that the nearly equal proportion of respondents had low (50.3) and high (49.7%) level of PWB with individual scores ranging from 147 to 234.

**Table 2:** Respondents’ level of psychological wellbeing *N* = 300.

Level of PWB	Frequency (n)	Percent (%)
Low	151	50.3
High	149	49.7
Minimum to Maximum Scores	147–234	
Median, IQR	194 (Q1 = 183.25, Q3 = 206.00)	

*Note.* Low = ≤ Median Value (i.e., 194.0), and High PWB = > Median Value (> 194) based on categorization in prior literature by Fergus et al. (2024).

### Socio-demographic, financial, and health-related factors associated with psychological wellbeing

In the bivariable logistic regression analysis, sex, marital status, offspring status, literacy status, former employment status, pension status, and receiving OAA were significantly associated with PWB (Table 3). Male older adults had higher odds of high PWB compared with females (*COR* = 2.13, 95% *CI*: 1.34–3.38, *p* = .001) (Table 3). Similarly, higher odds of high PWB were observed among married older adults living with a spouse (*COR* = 2.48, 95% *CI*: 1.54–3.99, *p* < .001), literate respondents (*COR* = 3.47, 95% *CI*: 2.09–5.77, *p* < .001), those ever employed (*COR* = 5.68, 95% *CI*: 2.87–11.22, *p* < .001), pension recipients (*COR* = 4.32, 95% *CI*: 1.90–9.80, *p* < .001), respondents who had offspring (*COR* = 5.78, 95% *CI*: 1.26–26.52, *p* = .024) (Table 3). In contrast, older adults receiving OAA had lower odds of high PWB than those not receiving OAA (*COR* = 0.38, 95% *CI*: 0.24–0.61, *p* < .001). However, age group, type of family, current employment status, receiving financial support from offspring, presence of chronic disease, perceived overall health status, and ability to perform activities of daily living were not significantly associated with PWB in the bivariable analysis (*p* > .05) (Table 3).

**Table 3:** Factors associated with respondents’ level of psychological wellbeing *N* = 300.

Variables	Level of PWB		*p*-value	*COR* [95% *CI*]	*AOR* [95% *CI*]	*p*-value
	Low n (%)	High n (%)				
**Age Group (In Completed Years) ^a^**						
≤ 72 ^b^	70 (45.8)	83 (54.2)	.106	.69[.44, 1.08]	.1.44 [.74, 2.81]	.286
>72	81 (55.1)	66 (44.9)				
**Sex ^a^**						
Female ^b^	94 (59.1)	65 (40.9)				
Male	57 (40.4)	84 (59.6)	.001**	2.13 [1.34, 3.38]	1.10 [.62, 1.97]	.740
**Marital Status ^a^**						
Never Married/Widowed/Separated ^b^	77 (63.6)	44 (36.4)				
Married and living with Spouse	74 (41.3)	105 (58.7)	<.001***	2.48 [1.54, 3.99]	1.75 [1.02, 3.02]	.044*
**Had Offspring**						
Yes	140 (48.8)	147 (51.2)	.024*	5.78 [1.26, 26.52]	Not Included	
No^b^	11 (84.6)	2 (15.4)				
**Literacy Status ^a^**						
Illiterate ^b^	72 (69.9)	31 (30.1)				
Literate	79 (40.1	118 (59.9)	<.001***	3.47 [2.09, 5.77]	2.12 [1.18, 3.82]	.013*
**Type of Family**						
Nuclear ^b^	28 (49.1)	29 (50.9)				
Joint and Extended	123 (50.6)	120 (49.4)	.839	.94 [.53, 1.68]	Not Included	
**Former Employment Status ^a^**						
Never employed ^b^	139 (58.2)	100 (41.8)				
Ever employed	12 (19.7)	49 (80.3)	<.001***	5.68 [2.87, 11.22]	3.94 [1.36, 11.36]	.011*
**Receiving Pension (Social Security Benefit from Employers) ^a^**						
Yes	8 (21.6)	29 (78.4)	<.001***	4.32 [1.90, 9.80]	.64 [.16, 2.49]	.518
No^b^	143 (54.4)	120 (45.6)				
**Current Employment Status**						
Employed	2 (25.0)	6 (75.0)	.167	3.13 [.62, 15.74]	Not Included	
Not employed ^b^	149 (51.0)	143 (49.0)				
**Receiving Financial Support from Offspring**						
Yes ^b^	104 (52.3)	95 (47.7)	.349	.80 [.49, 1.29]	Not Included	
No	47 (46.5)	54 (53.5)				
**Presence of Chronic Disease**						
Yes	105 (49.8)	106 (50.2)	.761	.1.08 [.66, 1.77]	Not Included	
No^b^	46 (51.7)	43 (48.3)				
**Perceived Overall Health Status**						
Very Poor and Fair^b^	100 (52.4)	91 (47.6)				
Good and Very Good	51 (46.8)	58 (54.1)	.354	1.25 [.78, 2.00]	Not Included	
**Ability to Perform ADLs**						
Yes	147 (49.8)	148 (50.2)	.215	4.03 [.45, 36.46]	Not Included	
No^b^	4 (80.0)	1(20.0)				
**Receiving old age allowance (Social Security Benefit) from the Government ^a^**						
Yes	104 (60.5)	68 (39.5)	<.001***	.38 [.24, .61]	.47 [.23, .96]	.037*
No^b^	47 (36.7)	81 (63.3)				

*Note.* ^a^: Variables included in the multivariable logistic model. ^b^: Reference Category. No multicollinearity: Based on correlation statistics: .00 to .72; Tolerance ranged between: .42 and .82, Variance Inflation Factor: 1.22 to 2.29. Omnibous Tests of Model Coefficients: *χ²* (*df* = 7, *N* = 300) = 52.70, *p* < .001. Cox and Snell *R* Square = .161, Nagelkerke *R* Squared = .215. Hosmer and Lemeshow Goodness of Fit Test: *χ²* (*df* = 7, *N* = 300) = 10.67 *p* = .154. *COR*: Crude Odds Ratio. Variables Adjusted in multivariable binary logistic regression model: age group, sex, marital status, literacy status, former employment status, receipt of pension, and receipt of OAA. *AOR*: Adjusted Odds Ratio. *CI*: Confidence Interval. * = *p*-value significant at <.05 level (2-tailed). ** = *p*-value significant at <.01 level (2-tailed). *** = *p*-value significant at <.001 level (2-tailed).

In the multivariable logistic regression analysis, marital status, literacy status, former employment status, and receiving OAA remained significantly associated with PWB. Older adults who were married and living with a spouse (*AOR* = 1.75, 95% *CI*: 1.02–3.02, *p* = .044), literate (*AOR* = 2.12, 95% *CI*: 1.18–3.82, *p* = .013), and ever employed (A*OR* = 3.94, 95% *CI*: 1.36–11.36, *p* = .011) had higher odds of high PWB. Conversely, older adults receiving OAA had lower odds of high PWB compared with those not receiving OAA (*AOR* = 0.47, 95% *CI*: 0.23–0.96, *p* = .037). Age group, sex, and pension status were not significantly associated with PWB in the adjusted model (*p* > .05) (Table 3). The logistic regression model was statistically significant, χ² (7, N = 300) = 52.70, *p* < .001. The model explained between 16.1% (Cox & Snell *R²*) and 21.5% (Nagelkerke *R²*) of the variance in the outcome. The Hosmer–Lemeshow goodness-of-fit test indicated an adequate model fit, *χ²* (7) = 10.67, *p* = 0.154. In the adjusted model, marital status, literacy status, former em*p*loyment status, and receipt of OAA were factors independently associated with psychological well-being among older adults (Table 3).

## Discussion

This study utilized a cross-sectional analytical design involving 300 older adults, selected through non-probability quota sampling based on daycare centers. The discussion focuses on the associations between socio-demographic, financial, and health-related characteristics and level of PWB among older adults. Furthermore, the findings were compared with previous studies to identify consistencies and discrepancies, thereby offering insights into how the results correspond with or differ from existing literature, especially within Nepal’s socio-cultural context.

The present study reveals that marital status was significantly associated with PWB among older adults attending day care centers of Kathmandu. This finding is consistent with previous studies conducted in Spain [13], Nigeria [12], Bhutan [15,16], and Bangladesh [17]. A plausible explanation for this association is that married individuals are more likely to benefit from supportive and intimate relationships that provide emotional companionship, social support, and opportunities to share daily experiences and concerns [34]. Consequently, marital relationships may serve as a buffer against psychosocial problems such as loneliness, social isolation, and emotional distress, while promoting quality of life, PWB, and life satisfaction among older adults [34]. Similarly, literacy status was significantly associated with PWB among older adults attending daycare centers. This finding is consistent with studies conducted in Nigeria [12], Ghana [18], Bhutan [16], Bangladesh [17], India [20], and Nepal [19]. The observed association may be explained by the advantages associated with literacy and education, including greater access to health information, improved healthcare utilization, and enhanced emotional well-being throughout the life course [35]. Furthermore, higher educational attainment is often linked to better socioeconomic opportunities, such as increased income and employment, which may contribute to improved PWB and life satisfaction [35].

Furthermore, the current study shows that former employment status was significantly associated with level of PWB among older adults attending daycare centers. Similarly, studies from Nigeria [12], Ghana [18], and Nepal [19] reported higher PWB among those with a history of formal employment. This association can be explained by role theory, which emphasizes that socially defined roles provide structure, identity, and engagement with social networks [36]. In the Nepali context, many older adults rely on insufficient OAA or other non-contributory benefits to meet their basic needs, as formal social security systems remain limited. However, those with former employment may receive benefits such as pensions, gratuity, provident funds, or citizenship funds upon retirement. Consequently, employment during working age can provide financial security through these benefits and personal savings, thereby reducing economic dependence, alleviating stress, and promoting PWB. Thus, formal employment offers financial security and social benefits that buffer against economic stressors and enhance life satisfaction [19]. Beyond financial security, employment fosters self-reliance, confidence, autonomy, social recognition, purpose, and personal development [37,38]. Positive workplace interactions further strengthen well-being, while meaningful work aligned with personal values enhances identity, self-esteem, and sense of purpose in life [38–40]. Furthermore, stable employment across the life course provides cumulative advantages, including access to resources, healthcare, and social protection, which reduce mental health burdens in later life [41]. Taken together, these pathways help explain the observed association between former employment status and PWB, highlighting the long-term psychosocial benefits of labor force participation.

In contrast, the present study reveals that older adults receiving the OAA had lower odds of high PWB compared to those not receiving the allowance. Previous studies in Nepal have reported several benefits of social security assistance among older adults. Sedhai [42] highlighted that financial assistance can empower older adults and enhance their overall quality of life. Similarly, OAA has been associated with increased social participation by enabling older adults to form new friendships, engage in social activities, and strengthen their sense of belonging and importance within their social networks [43]. Beneficiaries may also perceive the allowance as a form of governmental support, which can enhance self-respect, increase social recognition from neighbors, reduce financial dependence on family members, and support essential expenditures such as food, healthcare, and clothing [43]. Despite these potential benefits, PWB in later life is a multidimensional construct influenced by factors beyond financial support alone. Well-being is shaped by functional ability, which emerges from the interaction between an individual’s intrinsic capacity and their environment [44]. Additionally, social relationships, societal attitudes and values, health and social policies, and the availability of appropriate services all contribute to older adults’ well-being [44]. Therefore, the observed association may reflect broader socioeconomic, health, and functional vulnerabilities among allowance recipients rather than a direct effect of the allowance itself. Therefore, further research is warranted to explore the underlying mechanisms of this association and to identify strategies to enhance psychological well-being among older adults attending daycare centers.

On the other hand, age group, sex, offspring status, family type, pension receipt, financial support from offspring, presence of chronic disease, and perceived overall health status were not significantly associated with PWB among older adults attending daycare centers. However, previous studies have reported significant associations of age [12–15], sex [12,16–19], financial resources, family structure, living arrangements, perceived health, comorbidities, and supportive family relationships [13–19,21] with PWB. Several factors may explain these differences. First, previous studies often examined related constructs such as stress [14], distress [17], or depression [19], rather than PWB. In addition, differences in measurement tools and analytical methods may have contributed to variations in findings [12,15,16]. Second, many previous studies were conducted among older adults residing in old-age homes [13] or community settings [12,13,15–19], whereas the present study focused on daycare center attendees. Daycare centers may enhance PWB through social engagement, companionship, structured activities, and supportive services that reduce loneliness and promote well-being [45]. Consistent with this view, the present findings suggest that psychosocial benefits provided by daycare centers may contribute to more stable well-being across different sociodemographic and health groups. Moreover, PWB among daycare center attendees may be more strongly influenced by modifiable psychosocial resources, such as self-efficacy, resilience, perceived social support, social connectedness, spirituality, and reduced loneliness [22]. Collectively, these findings suggest that social, relational, and employment-related factors including marital status, literacy status, former employment, and receipt of OAA play a more important role in shaping PWB than non-modifiable characteristics such as age, sex, and family type.

Several limitations should be considered when interpreting the findings of this study. First, the cross-sectional design does not allow causal inferences between PWB and its associated factors. Therefore, the observed associations of marital status, literacy status, former employment, and OAA with PWB should not be interpreted as causal relationships. Second, the study was conducted only among older adults attending selected daycare centers in Kathmandu. Respondents were selected using a non-probability quota sampling method based on availability, which may introduce selection bias, and the number of respondents varied across centers. Therefore, the sample may not be fully representative of all older adults attending daycare centers in Kathmandu. In addition, current employment status may influence access to and utilization of daycare services; consequently, employed and unemployed older adults may be represented differently in the study sample. Future studies should examine the relationship between employment status and daycare service utilization. Furthermore, the sample comprised only older adults attending daycare centers, who may differ from those living in community or rural settings. As a result, the findings have limited generalizability to the broader older adult population in Nepal.

Third, the data were based on self-reported information, which may be subject to recall and social desirability bias. Key variables such as marital status, literacy status, etc. were also categorized into broad groups due to small numbers in some categories. While this approach was necessary for statistical stability, it may have reduced variability and limited the ability to capture more detailed differences. In addition, clustering of participants within daycare centers was not fully accounted for in the analysis, which may have slightly affected the precision of the estimates. Finally, PWB is influenced by multiple factors beyond OAA and the sociodemographic variables included in this study, including cultural, environmental, and life-course experiences that were not fully examined. Future research using longitudinal designs, more representative sampling, and multilevel analytical approaches is recommended to better understand the role of OAA, marital status, literacy status, former employment, and other factors associated with PWB among older adults.

## Conclusions

Marital status, literacy status, former employment, and receipt of the OAA were significantly associated with PWB. Notably, OAA recipients showed lower odds of high PWB, suggesting that financial support alone may not be sufficient to ensure better PWB in later life. These findings highlight the need for a more holistic approach to promoting PWB among older adults. In addition to financial assistance, targeted interventions should focus on older adults who are illiterate, those who are divorced, separated, unmarried, or widowed, and those with no formal employment history.

## Supporting information

S1 Table Association between different independent variables and level of psychological wellbeing. (PDF)

S2 Table Correlations of different independent variables with PWB. (PDF)

S3 Table Clustering effect assessment (One-way ANOVA Approach). (PDF)

S4 File Sample of informed consent form. (PDF)

## Abbreviations

ADLs: Activities of Daily Living
AOR: Adjusted Odds Ratio
CI: Confidence Interval
COR: Crude Odds Ratio
EPV: Events-per-variable
PWB: Psychological Wellbeing
RA: Research Assistants
SD: Standard Deviation
