# Supplementary material for: Psychological wellbeing and its associated factors among older adults attending daycare centers in Kathmandu, Nepal: A cross-sectional study
Source: PLoS One. 2026 Jul 15;21(7):e0353748. doi: 10.1371/journal.pone.0353748 (PMC13372132; doi:10.1371/journal.pone.0353748)
Supplement: S1 Table — (PDF) [file pone.0353748.s001.pdf]

S1 Table. Association Between Different Independent Variables and Level of Psychological Wellbeing

N = 300

**Variables**	**Level of PWB**		***p*-value**	X^2	***COR* [95% *CI*]**
	**Low**	**High**			
	**n (%)**	**n (%)**			
**Age Group (In Completed Years)**					
≤ 65	15 (41.7)	21 (58.3)	.279	3.84	
65 to 74	65 (48.1)	70 (51.9)			
75 to 84	57 (52.8)	51 (47.2)			
>85	14 (66.7)	7 (33.3)			
**Sex**					
Female	94 (59.1)	65 (40.9)	<.001***	10.45	2.13 [1.34, 3.38]
Male	57 (40.4)	84 (59.6)			
**Marital Status [a]**					
Married and Living with Spouse	74 (41.3)	105 (58.7)	.007**	16.08	
Widow	50 (65.8)	26 (34.2)			
Widower	18 (58.1))	13 (41.9)			
Separated	7 (58.3)	5 (41.7)			
Unmarried	1 (100.0)	0 (0.0)			
Divorce	1 (100.0)	0 (0.0)			
**Had Offspring**					
Yes	11 (84.6)	2 (15.4)	.011*	6.39	5.78 [1.26, 26.52]
No	140 (48.8)	147 (51.2)			
**Literacy Status**					
Illiterate	72 (69.9)	31 (30.1)	<.001	24.03	3.47 [2.09, 5.77]
Literate	79 (40.1)	118 (59.9)			

Note. [a]: Expected Cell Count Less than 5.

S1 Table. Association Between Different Independent Variables and Level of Psychological Wellbeing Cont'd

N = 300

**Variables**	**Level of PWB**		***p*-value**	***X^2***	***COR* [95% *CI*]**
	**Low**	**High**			
	**n (%)**	**n (%)**			
**Educational Level [a]**					
Illiterate	72 (69.9)	31 (30.1)	<.001***	31.93	
Read and Write Only	44 (49.4)	45 (50.6)			
Primary	15 (39.5)	23 (60.5)			
Lower Secondary	2 (50.0)	2 (50.0)			
Secondary	4 (22.2)	14 (77.8)			
Higher Secondary	9 (28.1)	23 (71.9)			
Bachelor and above	5 (31.2)	11 (68.8)			
**Type of Family**					
Nuclear	28 (49.1)	29 (50.9)	.653	.853	
Joint	118 (51.3)	112 (48.7)			
Extended	5 (38.5)	8 (61.5)			
**Former Employment Status**					
Never employed	139 (58.2)	100 (41.8)	<.001***	28.80	5.68 [2.87, 11.22]
Ever employed	12 (19.7)	49 (80.3)			
**Receiving Pension (Social Security Benefit from Employers)**					
Yes	8 (21.6)	29 (78.4)	<.001***	13.92	4.32 [1.90, 9.8)
No	143 (54.4)	120 (45.6)			
**Current Employment Status [a]**					
Employed	2 (1.3)	6 (75.0)	.146	2.11	3.13
Not employed	149 (51.0)	143 (49.0)			

Note. [a]: Expected Cell Count Less than 5.

S1 Table. Association Between Different Independent Variables and Level of Psychological Wellbeing Cont'd

N = 300

**Variables**	**Level of PWB**		***p*-value**	***X*²**	***COR* [95% *CI*]**
	**Low**	**High**			
	**n (%)**	**n (%)**			
**Receiving Financial Support from Offspring**					
Yes	104 (52.3)	95 (47.7)	.349	.879	.80 [.49, 1.29]
No	47 (46.5)	54 (53.5)			
**Presence of Chronic Disease**					
Yes	105 (49.8)	106 (50.2)			
No	46 (51.7)	43 (48.3)	.761	.093	1.08 [.66, 1.77]
**Perceived Overall Health Status** [a]					
Very Poor	5 (100.0)	0 (0.0)	.032*	10.52	
Poor	14 (51.9)	13 (48.1)			
Fair	81 (50.9)	78 (49.1)			
Good	48 (51.1)	46 (48.9)			
Very Good	3 (20.0)	12 (80.0)			
**Ability to Perform ADLs** [a]					
Yes	147 (49.8)	148 (50.2)	.181	1.79	4.03 [.45, 36.46]
No	4 (80.0)	1 (20.0)			
**Receiving old age allowance (Social Security Benefit) from the Government**					
Yes	104 (60.5)	68 (39.5)	<.001***	16.55	.38 [.24, .61]
No	47 (36.7)	81 (63.3)			

Note. [a]: Expected Cell Count Less than 5.
