# Supplementary material for: Psychological wellbeing and its associated factors among older adults attending daycare centers in Kathmandu, Nepal: A cross-sectional study
Source: PLoS One. 2026 Jul 15;21(7):e0353748. doi: 10.1371/journal.pone.0353748 (PMC13372132; doi:10.1371/journal.pone.0353748)
Supplement: S2 Table — (PDF) [file pone.0353748.s002.pdf]

S2 Table. Correlations of Different Independent Variables with PWB

N = 300

Variables	1	2	3	4	5	6	7	Tolerance	VIF
1. Age [a]	1							.59	1.71
2. Sex [b] (0 = female, 1 = male)	.09	1						.71	1.41
3. Marital Status [b] (1 = married and living with spouse, 0 = unmarried/divorced/widowed/separated)	.26***	-.25**	1					.82	1.22
4. Literacy Status [b] (0 = illiterate, 1 = literate)	-.14*	.41***	-.16***	1				.74	1.34
5. Former Employment Status (1 = employed, 0 = unemployed)	-.03	.30***	-.!5*	.35***	1			.44	2.29
6. Receiving Pension [b] (Social Security Benefit from Employers) (1 = yes, 0 = no)	.00	.24***	-.08	.23***	.72***	1		.42	1.40
7. Receiving Old Age Allowances [b] (1 = yes, 0 = no)	.64***	-.03	.24***	-.23***	-.32***	-.37***	1	.49	2.05
8. PWB	-.16**	.23***	-.21***	.32***	.18**	.18**	-.26***		

Note. [a]: Pearson Product Moment Correlation. [b]: Point Biserial Correlation.
