# Supplementary material for: Psychological wellbeing and its associated factors among older adults attending daycare centers in Kathmandu, Nepal: A cross-sectional study
Source: PLoS One. 2026 Jul 15;21(7):e0353748. doi: 10.1371/journal.pone.0353748 (PMC13372132; doi:10.1371/journal.pone.0353748)
Supplement: S3 Table — (PDF) [file pone.0353748.s003.pdf]

S3 Table: Clustering Effect Assessment (One-way ANOVA Approach)

Parameter	Value	Method	Interpretation
Study design	Cross-sectional	Study design	Single time-point data
Number of clusters (daycare centers)	6	Sampling structure	Small number of clusters
Total sample size	300	Study data	—
Average cluster size (k)	50	Average Cluster Size = [Total Sample Size (N)/ Number of Clusters (G)	Large cluster size
BMS (Between Groups Mean Square)	544.158	One-way ANOVA	—
WMS (Within Groups Mean Square)	287.635	One-way ANOVA	—
Intra-class Correlation [ICC] (ANOVA-based)	0.018	ICC = (BMS -WMS)/ [BMS + (k -1) WMS] (Shrout & Fleiss, 1979)	Low clustering effect
Design Effect (DEFF)	1.88	1 + (k−1) ICC (Alimohamadi & Sepandi, 2019)	Moderate variance inflation

Clustering Effect Assessment

The study included 6 daycare centers with a total sample of 300 participants, resulting in an average cluster size of 50. The ANOVA-based intraclass correlation coefficient (ICC) was 0.018, indicating low intra-center correlation among participants. The design effect was 1.88, suggesting moderate variance inflation due to clustering. Overall, clustering effects were present but weak.

References

Alimohamadi Y, Sepandi M. Considering the design effect in cluster sampling. J Cardiovasc Thorac Res. 2019;11(1):78. doi: 10.15171/jcvtr.2019.

Shrout, Patrick E.; Fleiss, Joseph L. (1979). Intraclass correlations: Uses in assessing rater reliability. Psychological Bulletin, 86(2), 420–428. doi:10.1037/0033-2909.86.2.420

Coding Scheme

For logistic regression analysis, all variables were ultimately coded into dichotomous forms. Socio-demographic variables included age group (0 = ≤72 years, 1 = >72 years; categorized based on the median age), sex (1 = male, 0 = female), marital status (1 = married and living with spouse, 0 = unmarried/divorced/widowed/separated), having offspring (1 = yes, 0 = no), literacy status (1 = literate, 0 = illiterate), and type of family (1 = joint/extended, 0 = nuclear). In addition, financial-related variables were included, such as former and current formal employment status (1 = employed, 0 = unemployed), receiving pension or social security benefit from employers (1 = yes, 0 = no), receiving financial support from offspring (1 = yes, 0 = no), and receiving the government OAA (1 = yes, 0 = no). Health-related variables were also considered, including the presence of chronic disease (1 = yes, 0 = no), perceived physical health status (1 = good/very good, 0 = very poor/poor/fair), and the ability to perform activities of daily living (ADLs) (1 = yes, 0 = no). Finally, the dependent variable, level of PWB, was dichotomized into high PWB (1) and low PWB (0), based on previously established categorization criteria [Fergus et al., 2018].
