# Supplementary material for: Psychological wellbeing and its associated factors among older adults attending daycare centers in Kathmandu, Nepal: A cross-sectional study
Source: PLoS One. 2026 Jul 15;21(7):e0353748. doi: 10.1371/journal.pone.0353748 (PMC13372132; doi:10.1371/journal.pone.0353748)
Supplement: S4 File — (PDF) [file pone.0353748.s004.pdf]

Annex V-1: Informed Consent Form for Structured Interview

Main Study Title: Factors Associated with Psychological Wellbeing among Older Adults of Kathmandu, Nepal: A Cross-Sectional Path-Analytical Study

Quantitative Descriptive Cross-Sectional Path-analytical Research Design. This design aims to identify the factors associated with psychological well-being among older adults in Kathmandu, Nepal.

Namaskar Dear Participant,

Statement. This study is going to be conducted by Dr. Rekha Timalsina, Patan Academy of Health Sciences, School of Nursing and Midwifery, Sanepa, Lalitpur; and the team. This study is ethically approved by an ethical review board of Nepal Health Research Council, Ramsahapath, Kathmandu, Nepal. The research team has obtained permission from the authority of the Kathmandu Metropolitan City [KMC] and the respective authority of the daycare centers of KMC.

Purpose and methods. This is a quantitative descriptive cross-sectional study regarding the factors associated with psychological well-being among older adults in Kathmandu, Nepal.

Expected duration of the participation and frequency of contact. The interview session will be held for 45 to 60 minutes. The frequency of contact is only one time**.**

Benefits. Although this interview does not have a direct benefit immediately, I hope that this interview will help to reveal the associated factors of psychological well-being among Nepalese older adults. In addition, booklets will be provided for you that include information on osteoarthritis, diabetes, sleep disturbances and sleep promotion activities, hypertension, dementia, stress-relieving breathing exercises, and tips for healthy aging. By acknowledging your participation in the study, you will be benefitted from informal health education by research team/research assistants on the ways of promoting psychological well-being. Likewise, the blood pressure measurement of each older adult after the interview session will be done.

Risks. There are no foreseeable risks or harm or inconvenience to you as a participants in this study. However, if you feel any discomfort during interviewing session, you can freely communicate with the researcher. Then, the researcher along with you will seek a solution to your discomfort.

Payment/reimbursement. Since there are no risks or harms associated with this study, the research team has decided against providing any payments or reimbursements.

Voluntary participation/withdrawal. You are selected purposefully. However, your participation in this study is voluntary. If you can read and write, the researcher/research assistants will provide an informed consent form to you by informing you of all the details of the study procedure. Then, written informed consent will be taken from you. If you are not be able to read and write, you will be informed of every detail regarding this study by the researcher/research team. Then, verbal informed consent or thumbprint for written informed consent from you and written informed consent from the witness (who will be one of your family members) will be taken by using an informed consent form. Two similar informed consent forms will be provided to you, one form that should be returned to the researcher, and another form for you for future reading. You have the right to refuse to share your feelings and withdraw your participation at any time,

even if you consented to participate in the study, and your relationship with the researcher or research assistants will not be affected by this withdrawal.

Privacy, Anonymity, and Confidentiality. Your privacy will be maintained by taking an interview with you separately sitting away from the authority of the daycare center, your colleagues, and other people, i.e., a one-to-one approach in a calm and quiet place based on your choice and feasibility. Confidentiality of your information will be maintained by keeping the collected information confidential, reporting the aggregate data for research purposes only, and keeping data access to the researcher only by securing the data under the double-locks in the researcher's room. All the information provided by you will be treated with strictest confidentiality during analysis, reporting, and publication. I would like to assure you that I and my team will not use your name or other recognizable marks in the interview schedule and record of your interview. Your identity will be coded by number in the interview schedule and analysis. Additionally, the informed consent form will be kept separate from the interview schedule.

Study team and the contact details along with the Ethical Review Board [ERB]: You are freely to ask any questions about the study or about being a subject. If you have any questions about this research or its conduct, you may contact me: Dr. Rekha Timalsina, mobile no. 9841368888/ 01-4114194.

If you would like to talk to someone other than the researcher about (1) concerns regarding this study, (2) research participant rights, (3) research-related injuries, or other human subjects' issues, please contact Ms. Namita Ghimire (Research Officer), Nepal Health Research Council, Ethical Review Board, e-mail: ethicalreviewb@gmail.com, Telephone no. 4254220 (Ext no 125).

Use of data. The data will be analyzed and published in aggregate form by maintaining anonymity, confidentiality, and privacy.

Statement of Consent

The researcher has explained the above information to me and provided one copy of this form to me. I understand the study procedure and my involvement in this study. Furthermore, I have received answers to my queries regarding this study. Finally, I voluntarily consent to participate in this study.

<table>
<tr><td rowspan="2">Signature:</td><td colspan="2">Thumb Print (For Illiterate Participants)</td></tr>
<tr><td>Right</td><td>Left</td></tr>
<tr><td colspan="3">Name of Participant with Contact No.:</td></tr>
<tr><td colspan="3">Date:</td></tr>
</table>

**For the Witnesses (One of the family members or local guardians of an illiterate older adult)**	**For the Researcher**
Verbal informed consent from my parents has already been taken from the researcher or research assistants. The researcher/ research assistant also explained me every detail regarding this study. Therefore, I voluntarily consent to involve my parents as a study participant.	I have explained this study to the above participant, or participant with the witness, and have sought his/her, or their understanding for informed consent.
**Signature:**	**Signature of Researcher or Research Assistants:**
**Name of Witness with Contact No.:**	**Name of Researcher:**
**Relation:**	**Date:**
**Date:**	

Thank you for your Valuable Time. Wish you for your good health and a happy life.
